# TOP2A as a prognostic biomarker in small cell carcinoma of the esophagus: an integrated bioinformatics and immunohistochemical study

**DOI:** 10.3389/fmolb.2025.1640881

**Published:** 2025-07-22

**Authors:** Xiaolei Yin, Xiaopeng Li, Lili Mi, Jiaojiao Hou, Fei Yin

**Affiliations:** ^1^Department of Gastroenterology, The Fourth Hospital of Hebei Medical University, Shijiazhuang, Hebei, China; ^2^Medical Record Room, The Fourth Hospital of Hebei Medical University, Shijiazhuang, Hebei, China

**Keywords:** TOP2A, small cell carcinoma of the esophagus, prognostic biomarker, bioinformatics, immunohistochemistry

## Abstract

**Background:**

Small cell carcinoma of the esophagus (SCCE) is an infrequent but highly aggressive cancer with a poor prognosis. Given its low incidence, there is a lack of validated biomarkers to guide risk stratification and inform treatment decisions.

**Methods:**

We extracted Differentially expressed genes (DEGs) from the GSE111044 dataset using standard bioinformatics workflows. A network of interacting proteins was assembled to determine hub genes, and TOP2A and CDK1 were selected for immunohistochemical (IHC) validation in 76 SCCE tumor samples. IHC staining scores were analyzed for associations with clinicopathological features. Survival analysis was conducted using Kaplan–Meier estimations and Cox regression modeling to pinpoint independent prognostic factors. To further assess the clinical utility, TOP2A expression was combined with VALSG staging for risk stratification.

**Results:**

A comparison between SCCE and adjacent normal tissues revealed 1,202 DEGs. PPI network analysis highlighted two hub genes, TOP2A and CDK1, which IHC validated in 76 SCCE samples. High TOP2A expression was significantly associated with advanced TNM stage (p = 0.020) and deeper tumor invasion (p = 0.004). Multivariate Cox analysis identified high TOP2A expression (HR = 1.92, 95% CI: 1.30–2.82, p = 0.001) and VALSG stage (HR = 2.20, 95% CI: 1.07–4.50, p = 0.031) as independent predictors of prognosis. Time-dependent ROC analysis indicated that the AUCs for the VALSG stage alone were 0.626, 0.638, and 0.602 at 1-, 2-, and 3-year time intervals, respectively. TOP2A alone yielded slightly higher AUCs of 0.719, 0.632, and 0.676. Notably, the combination of TOP2A and VALSG provided the greatest predictive accuracy, achieving AUCs recorded at 0.721, 0.734, and 0.773 at the respective time points.

**Conclusion:**

This study suggests that TOP2A is a novel, independent prognostic biomarker in SCCE. When integrated with the VALSG staging system, TOP2A expression enhances risk stratification and may serve as a useful adjunct in clinical prognostication. These findings support its clinical utility while emphasizing the necessity for future studies to include prospective validation.

## 1 Introduction

Small cell carcinoma of the esophagus (SCCE) is an uncommon and highly aggressive cancer, representing under 2% of all cancer cases (Ji et al., 2020). First described in the 1950s (McKeown, 1952), SCCE is characterized by rapid progression, early metastasis, and an unfavorable prognosis, with an average survival time of 8–13 months despite treatment (Jeene et al., 2019). Owing to its histological similarity to small cell lung cancer (SCLC), approaches to therapy for SCCE are largely adapted from SCLC protocols, most commonly platinum-based chemotherapy (Liu D. et al., 2021). However, clinical responses are typically short-lived, and most patients relapse quickly, underscoring both the biological heterogeneity and therapeutic challenges specific to SCCE (Zhang Q. et al., 2024).

Current prognostic assessments for SCCE primarily rely on traditional clinicopathological factors, including tumor location, treatment modality, and staging systems such as the tumor-node-metastasis (TNM) and Veterans Administration Lung Study Group (VALSG) classifications (Deng et al., 2016; Daiko and Kato, 2020; Yin et al., 2025). However, their prognostic value has been inconsistently reported across studies, and neither system fully reflects the molecular heterogeneity underlying clinical outcomes. Given the rarity of SCCE, conducting prospective multi-institutional studies remains a challenge. In this context, leveraging publicly available transcriptomic datasets, coupled with targeted experimental validation, offers a feasible and cost-effective approach to identify novel biomarkers for risk stratification.

TOP2A (DNA topoisomerase II alpha) encodes a nuclear enzyme that is crucial for DNA replication, transcription, and chromatin remodeling (Lockwood et al., 2022; Zhou et al., 2021). Its overexpression has been documented in numerous cancers, which is commonly connected to high proliferative activity and poor clinical outcomes (Hasib, 2022; Jia et al., 2021; Meng et al., 2022). Emerging studies further suggest that TOP2A may contribute to multiple oncogenic pathways, including those involving MAPK, AKT/mTOR, and cell cycle signaling (Liu SL. et al., 2021; Zhang K. et al., 2024; Liu et al., 2024). However, its clinical relevance in SCCE has not yet been investigated.

To the best of our knowledge, this is the first exploratory study to suggest and validate TOP2A as a potential prognostic biomarker in SCCE using an integrative approach that combines bioinformatics analysis and experimental validation. By combining transcriptomic analysis with immunohistochemical validation, we demonstrated that TOP2A expression correlates with poor prognosis and enhances the prognostic performance of the traditional VALSG staging system in risk stratification.

## 2 Materials and methods

### 2.1 Public dataset acquisition and preprocessing

The GEO database (GSE111044) provided the sole publicly accessible transcriptomic data currently available for SCCE (Liu et al., 2018). The following criteria were used to include data: (1) human samples (*Homo sapiens*), (2) histologically confirmed SCCE, and (3) availability of at least three matched pairs of tumor and adjacent normal tissues. Data preprocessing was performed in R (version 4.1.1), including normalization using the limma package and dimensionality reduction using the factoextra package (Ritchie et al., 2015; Kassambara, 2016). Given that GSE111044 includes only three paired tumor and adjacent normal samples, the downstream analysis should be interpreted as exploratory, and the findings require further validation in larger cohorts.

### 2.2 Differential gene expression and enrichment analysis

The limma package was used to identify DEGs between tumor and normal tissues, with |log2FC| > 2 and p < 0.05 set as cutoff values. DEGs were visualized using volcano and heatmap plots generated with the ggplot2 and pheatmap packages. Functional enrichment was performed using the clusterProfiler package (Wu et al., 2021), incorporating both Gene Ontology (GO) and Gene Set Enrichment Analysis (GSEA) frameworks.

### 2.3 Construction of protein interaction networks and selection of hub genes

A network of protein-protein interactions was constructed via the STRING database, applying a confidence score threshold greater than 0.7 (Szklarczyk et al., 2023). The resulting network was visualized in Cytoscape (version 3.8.1) (Otasek et al., 2019), and key hub genes were prioritized using the cytoHubba plugin, based on both Maximal Clique Centrality and Degree scoring algorithms.

### 2.4 Patient cohort and clinical data collection

A retrospective cohort study involving 76 patients confirmed with SCCE from June 2011 to September 2020 was identified at the Fourth Affiliated Hospital of Hebei Medical University. The criteria for inclusion: (1) histopathologically confirmed diagnosis of SCCE, (2) availability of tumor tissue samples, and (3) complete clinicopathological and follow-up information. Patients with coexisting malignancies or incomplete tumor resection were excluded. The study was approved by the institutional review board, and patients were followed at 3-month intervals until death or the final follow-up on 1 October 2024.

### 2.5 Immunohistochemical staining and scoring

A total of 76 SCCE tumor samples preserved as formalin-fixed, paraffin-embedded (FFPE) sections were subjected to immunohistochemical (IHC) analysis to evaluate the protein expression of TOP2A and CDK1. Sections with a thickness of 4 μm were stripped of paraffin in xylene, rehydrated through a graded ethanol series, and subjected to heat-induced antigen retrieval in citrate buffer (pH 6.0). Endogenous peroxidase activity was blocked using 3% hydrogen peroxide for 10 min. Slides were then incubated overnight at 4°C with primary antibodies against TOP2A and CDK1 (dilution 1:400), followed by the application of secondary antibodies and visualization using a standard diaminobenzidine (DAB) detection system. Hematoxylin was applied for counterstaining. Staining evaluation was independently conducted by two experienced pathologists. Staining intensity was scored from 0 to 3, corresponding to negative (0), weak (1), moderate (2), and strong (3). The proportion of positively stained tumor cells was graded as follows: 1 (≤25%), 2 (26%–50%), 3 (51%–75%), and 4 (>75%). The two scores were combined to calculate a composite IHC score, which was then categorized into low (2–3), medium (4–5), and high (6–7) expression levels.

### 2.6 Correlation analysis between TOP2A and CDK1 expression

The association between TOP2A and CDK1 protein expression levels, as measured by IHC scores, was evaluated using Spearman’s rank test. Scatter plots displaying correlation coefficients and p-values were generated with the ggpubr package in R.

### 2.7 Survival and prognostic analysis

Kaplan–Meier curves were generated to compare overall survival (OS) across gene expression subgroups, and statistical significance was tested using the log-rank method. Prognostic variables were further evaluated through univariate and multivariate Cox analyses, implemented by the survival and forestplot R packages. Variables with a p-value <0.05 in the univariate Cox analysis were included in the multivariate model.

### 2.8 Integration with VALSG staging and stratification analysis

To determine whether TOP2A expression improves prognostic stratification beyond conventional clinical staging, patients were stratified into three subgroups according to the combination of VALSG stage (limited vs extensive) and TOP2A expression (low vs medium/high): (1) low-risk group: limited stage and low TOP2A expression, (2) medium-risk group: either limited stage with medium/high TOP2A or extensive stage with low TOP2A, and (3) high-risk group: extensive stage and medium/high TOP2A expression. Survival differences among the subgroups were evaluated through Kaplan-Meier analysis and the log-rank test. Time-dependent ROC curves at 1-, 2-, and 3-year time points were generated using the “timeROC” package in R to assess the discriminatory performance of individual and combined models.

### 2.9 Statistical analysis

Statistical analyses were performed using R software (v4.1.1). Group comparisons for categorical data employed chi-square tests, continuity-adjusted methods, or Fisher’s exact test where applicable. For continuous data, the Wilcoxon rank-sum test was applied. A p-value less than 0.05 (two-sided) was considered to indicate statistical significance.

## 3 Results

### 3.1 Quality control and sample clustering

**FIGURE 1:**
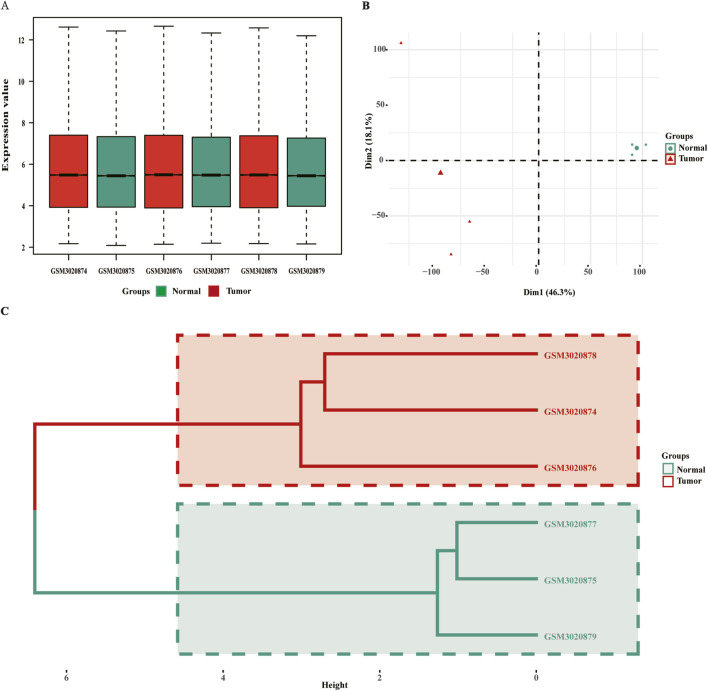
Quality control assessment of the GSE111044 dataset. **(A)** Boxplot showing normalized gene expression values for six samples; **(B)** Principal component analysis (PCA) plot based on gene expression data; **(C)** Hierarchical clustering tree showing sample relationships by expression similarity. PCA, principal component analysis.

The GSE111044 dataset comprised three matched samples of SCCE and adjacent normal tissues. As demonstrated by the boxplot (Figure 1A), gene expression distributions were consistent across all samples, suggesting successful normalization. Principal component analysis (PCA) and hierarchical clustering revealed clear segregation between tumor and normal samples (Figures 1B,C), supporting the dataset’s suitability for downstream differential expression analysis.

### 3.2 Differential gene identification and enrichment analysis

**FIGURE 2:**
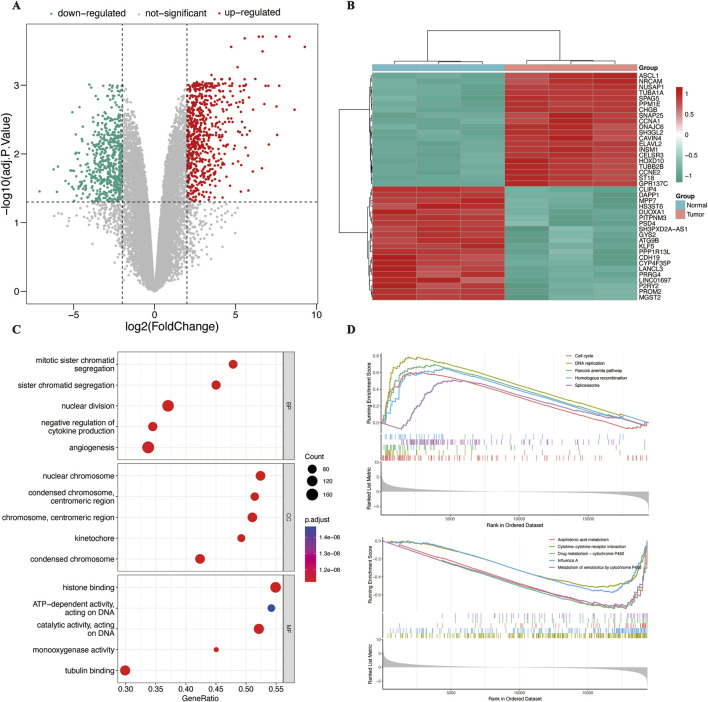
Differential gene expression and enrichment analysis. **(A)** Volcano plot showing upregulated (red) and downregulated (green) genes between SCCE and adjacent normal tissues; **(B)** Heatmap of the top 20 upregulated and 20 downregulated DEGs; **(C)** GO enrichment analysis of DEGs, displaying the top five terms in each of the BP, CC, and MF categories; **(D)** GSEA of DEGs showing the top five enriched pathways in the upregulated and downregulated gene sets. DEGs, differentially expressed genes; SCCE, small cell carcinoma of the esophagus; GO, Gene Ontology; BP, biological process; CC, cellular component; MF, molecular function; GSEA, Gene Set Enrichment Analysis.

There were 1,202 DEGs identified between SCCE and the adjacent normal tissues, comprising 656 upregulated and 546 downregulated genes. The overall pattern of DEGs was illustrated through a volcano diagram (Figure 2A), and the most significantly upregulated and downregulated 20 genes were visualized using a heatmap (Figure 2B).

To gain functional insights into these DEGs, GO and GSEA were performed, aiming to uncover their biological implications. The significantly enriched GO terms were categorized into three domains: CC, BP, and MF. CC terms were primarily associated with the nuclear chromosome, centromeric region, and condensed chromosome. MF terms involved histone binding, ATP-dependent DNA activity, catalytic activity, monooxygenase activity, and tubulin binding. BP terms were enriched in mitotic sister chromatid segregation, angiogenesis, and negative regulation of cytokine production (Figure 2C). GSEA was conducted to assess coordinated changes in signaling pathways. Upregulated gene sets were predominantly enriched in pathways related to the cell cycle, DNA replication, Fanconi anemia, homologous recombination, and the spliceosome. In contrast, downregulated gene sets were mainly associated with arachidonic acid metabolism, cytokine and receptor interactions, cytochrome P450 metabolism, and influenza A infection (Figure 2D).

### 3.3 Identification of hub genes based on PPI network analysis

A PPI network was generated using DEGs from the STRING database, with a confidence score greater than 0.7, to identify potential hub genes. To improve network clarity, unconnected or weakly linked nodes were excluded, resulting in a final network comprising 288 nodes and 3,142 edges.

**FIGURE 3:**
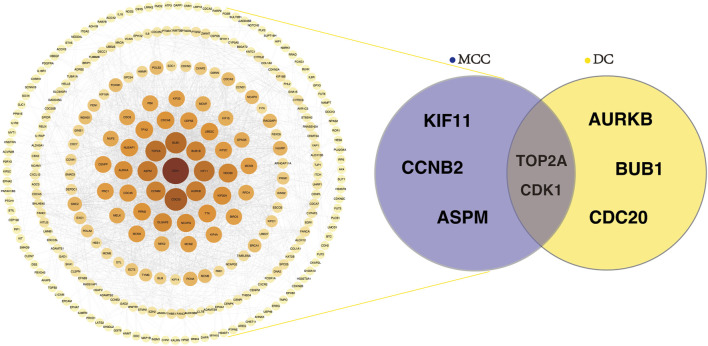
Identification of hub genes from the PPI network. Significant protein-protein interactions. Left: Visualization of the PPI network constructed from DEGs using a confidence score >0.7. Node color intensity indicates interaction strength, with darker nodes representing higher connectivity. Right: Venn diagram showing the overlap of the top five hub genes identified by MCC and DC algorithms, with TOP2A and CDK1 shared by both methods. PPI, protein–protein interaction; MCC, maximal clique centrality; DC, degree centrality.

Visualization and subsequent hub gene ranking were performed in Cytoscape (v3.8.1) using the cytoHubba extension, based on two network centrality algorithms: Maximal Clique Centrality and Degree. The top five genes identified by each method were intersected, revealing two overlapping hub genes, TOP2A and CDK1, which were considered the most central in the network (Figure 3).

### 3.4 Immunohistochemical expression and correlation analysis of TOP2A and CDK1 in SCCE

**FIGURE 4:**
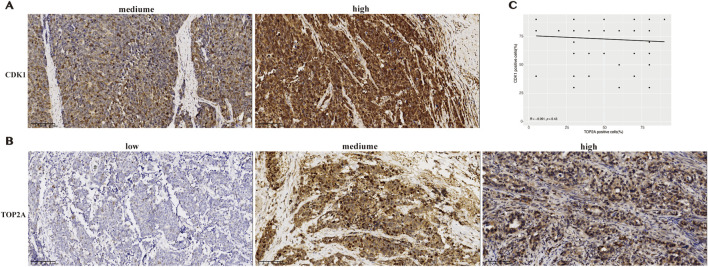
Immunohistochemical expression and correlation of CDK1 and TOP2A in SCCE. **(A)** Representative IHC images showing medium and high CDK1 expression in tumor tissues. Scale bar = 100 μm. **(B)** Representative IHC images showing low, medium, and high TOP2A expression in tumor tissues. Scale bar = 100 μm. **(C)** Scatter plot of Spearman correlation between CDK1 and TOP2A expression levels in SCCE tissues.

Immunohistochemical staining was performed to verify CDK1 and TOP2A protein expression in SCCE tissues. Among the 76 cases, CDK1 expression was classified as high in 62 (81.6%) and moderate in 14 (18.4%), with no samples showing low expression (Figure 4A). In contrast, TOP2A expression was high in 27 cases (35.5%), moderate in 35 (46.1%), and low in 14 (18.4%) (Figure 4B).

Correlation analysis revealed no statistically significant link between TOP2A and CDK1 protein expression levels (R = −0.091, p = 0.43) (Figure 4C).

### 3.5 Correlation of TOP2A and CDK1 expression with clinical and pathological characteristics

Based on immunohistochemical scores, CDK1 expression was classified into two levels: medium (4–5) and high (6–7), whereas TOP2A expression was divided into three groups: low (2–3), medium (4–5), and high (6–7). As summarized in Table 1, CDK1 expression showed no significant association with any clinicopathological parameters, including gender, age, tumor location, TNM stage, depth of invasion, lymph node status, or VALSG stage (all p > 0.05).

**TABLE 1:** Relationship of CDK1 expression and clinicopathologic characteristics in SCCE patients.

Clinicpathologic characteristics	N	CDK1 expression		$x^{2}$	*P*
		Medium	High		
Gender					
Male	48	8 (16.7%)	40 (83.3%)	0.267	0.760
Female	28	6 (21.4%)	22 (78.6%)		
Age					
<60	31	7 (22.6%)	24 (77.4%)	0.603	0.550
≥60	45	7 (15.6%)	38 (84.4%)		
Tumor Location					
Upper	6	2 (33.3%)	4 (66.7%)	1.284	0.552
Middle	31	5 (16.1%)	26 (83.9%)		
Lower	39	7 (17.9%)	32 (82.1%)		
TNM stage					
Ⅰ+Ⅱ	30	4 (13.3%)	26 (86.7%)	0.854	0.388
Ⅲ+Ⅳ	46	10 (21.7%)	36 (78.3%)		
Invasion depth					
T1+T2	30	4 (13.3%)	26 (86.7%)	0.854	0.388
T3+T4	46	10 (21.7%)	36 (78.3%)		
Lymph node metastasis					
Negative	26	5 (19.2%)	21 (80.8%)	0.000	1.000
Positive	50	9 (18.0%)	41 (82.0%)		
VALSG stage					
Limited	63	9 (14.3%)	54 (85.7%)	2.737	0.098
Extensive	13	5 (38.5%)	8 (61.5%)		

Abbreviations: TNM, stage, tumor-node-metastasis stage; VALSG, stage: Veterans Administration Lung Cancer Study Group stage.

In contrast, TOP2A expression showed a strong correlation with TNM stage (p = 0.020) and depth of invasion (p = 0.004) (Table 2). The proportion of patients with medium TOP2A expression increased with TNM stage, reaching 54.3% in stage III–IV, whereas only 33.3% of patients in stage I–II exhibited the same expression level. Similarly, medium and high expression levels were more frequently observed in the T3–T4 group (52.2% and 41.3%, respectively) than in the T1–T2 group (36.7% and 26.7%). No significant associations were found between TOP2A expression and the remaining clinical variables, including gender, age, tumor location, lymph node metastasis, or VALSG stage.

**TABLE 2:** Relationship of TOP2A expression and clinicopathologic characteristics in SCCE patients.

Clinicpathologic characteristics	N	TOP2A expression			$x^{2}$	*P*
		Low	Medium	High		
Gender						
Male	48	7 (14.6%)	25 (52.1%)	16 (33.3%)	2.247	0.329
Female	28	7 (25.0%)	10 (35.7%)	11 (39.3%)		
Age						
<60	31	7 (22.6%)	12 (38.7%)	12 (38.7%)	1.254	0.507
≥60	45	7 (15.6%)	23 (51.1%)	15 (33.3%)		
Tumor Location						
Upper	6	2 (33.3%)	3 (50.0%)	1 (16.7%)	5.842	0.185
Middle	31	6 (19.4%)	10 (32.3%)	15 (48.3%)		
Lower	39	6 (15.4%)	22 (56.4%)	11 (28.2%)		
TNM stage						
Ⅰ+Ⅱ	30	10 (33.3%)	10 (33.3%)	10 (33.3%)	7.792	0.020
Ⅲ+Ⅳ	46	4 (8.7%)	25 (54.3%)	17 (37.0%)		
Invasion depth						
T1+T2	30	11 (36.7%)	11 (36.7%)	8 (26.7%)	11.001	0.004
T3+T4	46	3 (6.5%)	24 (52.2%)	19 (41.3%)		
Lymph node metastasis						
Negative	26	7 (26.9%)	9 (34.6%)	10 (38.5%)	2.788	0.254
Positive	50	7 (14.0%)	26 (52.0%)	17 (34.0%)		
VALSG stage						
Limited	63	14 (22.2%)	30 (47.6%)	19 (30.2%)	5.784	0.055
Extensive	13	0 (0.0%)	5 (38.5%)	8 (61.5%)		

Abbreviations: TNM, stage, tumor-node-metastasis stage; VALSG, stage: Veterans Administration Lung Cancer Study Group stage.

### 3.6 Prognostic significance of TOP2A and VALSG stage in SCCE

**FIGURE 5:**
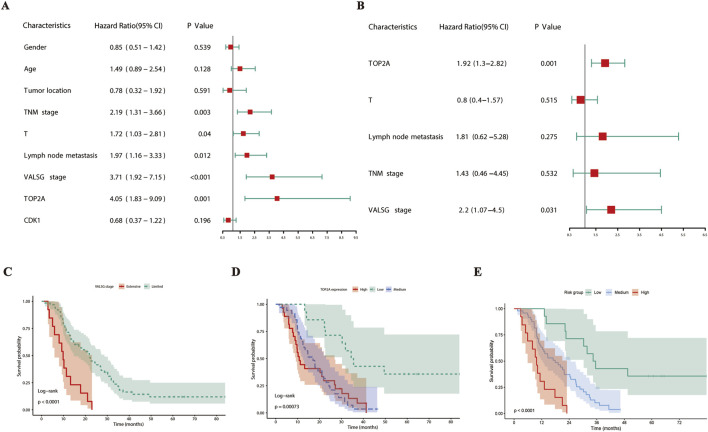
Prognostic impact of TOP2A expression and VALSG stage in SCCE patients. **(A)** Forest plot of univariate Cox regression analysis for overall survival. **(B)** Forest plot of multivariate Cox regression analysis identifying independent prognostic factors. **(C)** Kaplan–Meier survival curves comparing overall survival between VALSG limited and extensive stages. **(D)** Kaplan–Meier survival curves stratified by TOP2A expression levels (low, medium, high). **(E)** Kaplan–Meier survival curves showing integrated risk stratification based on VALSG stage and TOP2A expression levels.

To determine which clinicopathological features independently influenced overall survival in SCCE, we applied multivariate Cox regression analysis. The evaluation included variables such as age, gender, tumor location, TNM stage, T stage, lymph node metastasis, VALSG stage, and expression levels of TOP2A and CDK1. Univariate Cox analysis identified TNM stage (p = 0.003), T (p = 0.040), lymph node metastasis (p = 0.012), VALSG stage (p < 0.001), and TOP2A expression (p = 0.001) as variables that were significantly related to overall survival (Figure 5A). In multivariate analysis, only TOP2A expression (HR = 1.92, 95% CI: 1.30–2.82, p = 0.001) and VALSG stage (HR = 2.20, 95% CI: 1.07–4.50, p = 0.031) remained statistically significant predictors (Figure 5B). Conversely, CDK1 expression showed no prognostic relevance in either univariate or multivariate models.

Kaplan-Meier analysis showed that patients diagnosed with extensive-stage disease exhibited markedly reduced overall survival compared to those with limited-stage tumors (p < 0.0001, Figure 5C). Similarly, medium or high TOP2A expression was linked to worse survival outcomes than low levels (p = 0.00073, Figure 5D). Notably, integration of VALSG staging and TOP2A expression enabled the definition of three prognostic categories, among which patients classified as high risk experienced the poorest survival (p < 0.0001, Figure 5E).

### 3.7 Prognostic performance of combined TOP2A expression and VALSG stage

**FIGURE 6:**
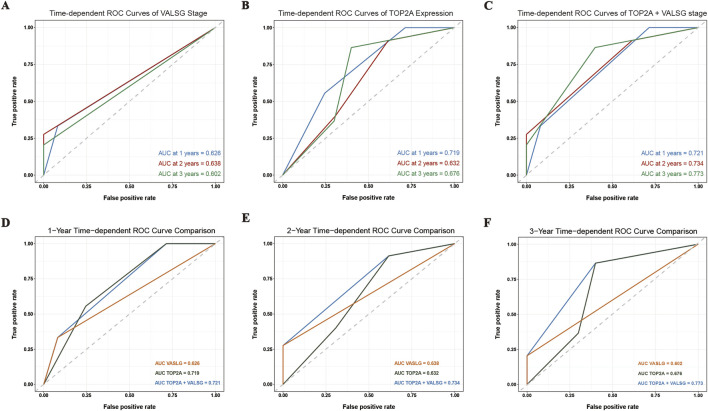
Time-dependent ROC analysis comparing the prognostic performance of VALSG stage, TOP2A expression, and their combination in SCCE. **(A–C)** 1-, 2-, and 3-year ROC curves based on VALSG stage, TOP2A expression, and their combination, respectively. **(D–F)** Comparative ROC curves illustrating the predictive accuracy of VALSG stage, TOP2A expression, and the combined model at 1, 2, and 3 years, respectively. ROC, receiver operating characteristic; AUC, area under the curve.

To further assess the prognostic value of TOP2A expression and VALSG stage, time-dependent ROC analyses were performed at 1-, 2-, and 3-year time points. As shown in Figures 6A–C, the AUCs for VALSG stage alone were 0.626, 0.638, and 0.602, indicating limited predictive accuracy. TOP2A alone yielded slightly higher AUCs of 0.719, 0.632, and 0.676 (Figures 6D–F). Notably, the combined approach integrating TOP2A expression with VALSG stage demonstrated the best performance, with AUC values reaching 0.721, 0.734, and 0.773 at the first, second, and third year survival time points, respectively. These results suggest that incorporating TOP2A into the conventional VALSG staging system improves prognostic discrimination and may enhance risk stratification in patients with SCCE.

## 4 Discussion

This research suggests that TOP2A may serve as a standalone prognostic marker for SCCE, based on an integrative approach combining bioinformatics analysis and immunohistochemical validation. Although the VALSG stage has long served as a clinical stratification tool in SCCE management (1), our findings suggest that its prognostic accuracy is limited when applied alone. We demonstrated that the expression of TOP2A is significantly correlated with overall survival, and that combining TOP2A expression with VALSG staging enhances its predictive performance, as supported by multivariate Cox regression and time-dependent ROC analysis. These results underscore the potential clinical utility of TOP2A as both an individual prognostic factor and an adjunct to conventional staging systems, contributing to more refined risk stratification in this rare and understudied malignancy.

Univariate and multivariate Cox models repeatedly confirmed TOP2A as an independent predictor of prognosis in SCCE. Kaplan-Meier analysis demonstrated that patients exhibiting medium to high TOP2A levels experienced notably reduced survival compared to those with low expression. Immunohistochemical analysis further supported these findings, revealing a high frequency of TOP2A positivity in tumor tissues. Moreover, elevated TOP2A levels were linked to a higher TNM stage and deeper tumor invasion, suggesting a potential role in tumor aggressiveness. By contrast, although CDK1 was overexpressed at the transcriptomic level, it showed no significant association with survival or clinicopathological features in our cohort. CDK1 was included in this study due to its centrality in the PPI network derived from differentially expressed genes. However, it did not show a significant association with clinicopathological features or overall survival in the IHC validation cohort. This result underscores that network centrality does not necessarily translate into clinical relevance, highlighting the complexity of biological systems and the need for empirical validation.

Aberrant overexpression of TOP2A has been widely reported in multiple cancers, such as liver cancer and gastric cancer, where it is linked to aggressive tumor behavior and poor prognosis (Meng et al., 2022; Mangalaparthi et al., 2023; Mokhlesi et al., 2023). To date, however, the potential prognostic role of TOP2A in SCCE remains unexplored. Our findings provide initial evidence that TOP2A may serve as a clinically relevant biomarker in this rare tumor type, with potential utility as both a molecular classifier and a tool for risk stratification in future SCCE management.

Although VALSG staging remains a widely used clinical classification system in SCCE, its prognostic performance appears suboptimal (Rice et al., 2017). In our cohort, VALSG stage alone was associated with only modest time-dependent AUC values. By contrast, TOP2A expression exhibited greater predictive power, particularly at earlier time points. Notably, the combined assessment of TOP2A expression and VALSG stage improved prognostic accuracy across all time points, as demonstrated by higher AUCs in time-dependent ROC analyses. Importantly, this improvement appeared to result from the independent prognostic contributions of TOP2A and VALSG stage, rather than a statistical interaction. Thus, the combination should be interpreted as an additive model. These findings suggest that integrating molecular features such as TOP2A expression may address the limitations of conventional staging and enhance clinical risk stratification in SCCE. In our study, TNM stage did not remain statistically significant in multivariate analysis, whereas VALSG stage and TOP2A expression emerged as independent prognostic factors. This observation led us to evaluate their combined performance, which yielded improved risk stratification. Given the strong associations between TOP2A expression and both TNM stage and tumor invasion depth, future studies could explore whether combining TOP2A with TNM staging may offer comparable or even superior prognostic value.

Such integrative approaches that combine molecular biomarkers with established clinical staging systems have demonstrated prognostic value across multiple cancer types, including breast cancer, oral cancer, and hepatocellular carcinoma (Vissio et al., 2020; Lee et al., 2020; Borde et al., 2022). Extending this paradigm to rare malignancies, our findings indicate that a similar strategy may be applicable to SCCE. Given the pronounced clinical heterogeneity and absence of standardized prognostic models in this disease, incorporating TOP2A expression into the existing VALSG staging system may offer a more refined stratification framework to guide treatment decisions and inform patient counseling.

TOP2A encodes a nuclear enzyme essential for DNA replication, chromosome condensation, and cell cycle progression (Abbasi and Alexandrov, 2022; Pommier et al., 2022). Its overexpression has been consistently linked to high proliferative activity and aggressive tumor behavior across various malignancies (Wang et al., 2022; Wang et al., 2023; Hu et al., 2022). In our study, DEGs in SCCE were significantly enriched in pathways related to mitotic cell cycle regulation, chromosome segregation, and DNA repair, which are biological processes intimately associated with TOP2A function. Moreover, GSEA analyses revealed that upregulated genes of SCCE were predominantly associated with cell cycle progression, DNA replication, and homologous recombination, collectively indicating elevated replicative stress and genomic instability, which are hallmarks of aggressive tumor biology. Our results provide a robust biological rationale for the prognostic significance of TOP2A in SCCE, suggesting that it may have potential as a clinical prognostic biomarker in the future.

Given the rarity of SCCE and the current lack of disease-specific *in vitro* and *in vivo* models, future research should prioritize the development of appropriate experimental systems to elucidate the functional role of TOP2A. Additionally, increasing the sample size by collaborating across multiple centers and including transcriptomic-level validation could further substantiate the prognostic value of TOP2A. These efforts may ultimately facilitate the development of clinically actionable prognostic tools that integrate molecular markers, such as TOP2A, into conventional staging systems, thereby advancing personalized treatment approaches for patients with SCCE.

The study has several limitations that need to be acknowledged. First, the data analysis was based on a single dataset comprising only three paired SCCE and adjacent normal tissue samples, potentially limiting the robustness and generalizability of the identified DEGs. However, this dataset remains the only publicly available high-throughput transcriptomic resource specific to SCCE, emphasizing the urgent need for additional data generation. Second, due to the rarity of SCCE, only formalin-fixed, paraffin-embedded samples were available for immunohistochemical validation, thereby restricting the analysis to the protein level. The prospective collection of fresh-frozen tissues will be critical to confirm these findings at the transcriptomic level, such as through quantitative PCR or RNA *in situ* hybridization. Third, this study is a single-center retrospective study, which may limit the generalizability of the findings. Therefore, future validation in larger, prospective multicenter cohorts is warranted. Taken together, these limitations underscore the need for broader validation and mechanistic studies to fully establish the clinical relevance of TOP2A in SCCE.

## 5 Conclusion

To our knowledge, this is the first study to identify and validate TOP2A as a prognostic biomarker in SCCE using integrated bioinformatics and immunohistochemical approaches. Elevated TOP2A expression was associated with more advanced disease characteristics and independently predicted worse overall survival. Importantly, incorporating TOP2A expression into the VALSG staging system appeared to enhance risk stratification accuracy. These observations highlight the clinical potential of TOP2A not only as an independent prognostic biomarker but also as a valuable adjunct to traditional staging methods in SCCE. Further investigations are warranted to substantiate these findings and elucidate the molecular mechanisms underlying TOP2A in this rare malignancy.

## Data Availability

Publicly available datasets were analyzed in this study. This data can be found here: GEO database (https://www.ncbi.nlm.nih.gov/geo/), accession number GSE111044.
